# Synergistic Effect of Caffeine and Glucocorticoids on Expression of Surfactant Protein B (SP-B) mRNA

**DOI:** 10.1371/journal.pone.0051575

**Published:** 2012-12-14

**Authors:** Markus Fehrholz, Iliana Bersani, Boris W. Kramer, Christian P. Speer, Steffen Kunzmann

**Affiliations:** 1 Children’s Hospital, University of Wuerzburg, Wuerzburg, Germany; 2 A. Gemelli Hospital, Catholic University of the Sacred Heart, Rome, Italy; 3 Department of Pediatrics, University Hospital Maastricht, Maastricht, The Netherlands; Hôpital Robert Debré, France

## Abstract

Administration of glucocorticoids and caffeine is a common therapeutic intervention in the neonatal period, but possible interactions between these substances are still unclear. The present study investigated the effect of caffeine and different glucocorticoids on expression of surfactant protein (SP)-B, crucial for the physiological function of pulmonary surfactant. We measured expression levels of SP-B, various SP-B transcription factors including erythroblastic leukemia viral oncogene homolog 4 (ErbB4) and thyroid transcription factor-1 (TTF-1), as well as the glucocorticoid receptor (GR) after administering different doses of glucocorticoids, caffeine, cAMP, or the phosphodiesterase-4 inhibitor rolipram in the human airway epithelial cell line NCI-H441. Administration of dexamethasone (1 µM) or caffeine (5 mM) stimulated SP-B mRNA expression with a maximal of 38.8±11.1-fold and 5.2±1.4-fold increase, respectively. Synergistic induction was achieved after co-administration of dexamethasone (1 mM) in combination with caffeine (10 mM) (206±59.7-fold increase, p<0.0001) or cAMP (1 mM) (213±111-fold increase, p = 0.0108). SP-B mRNA was synergistically induced also by administration of caffeine with hydrocortisone (87.9±39.0), prednisolone (154±66.8), and betamethasone (123±6.4). Rolipram also induced SP-B mRNA (64.9±21.0-fold increase). We detected a higher expression of ErbB4 and GR mRNA (7.0- and 1.7-fold increase, respectively), whereas TTF-1, Jun B, c-Jun, SP1, SP3, and HNF-3α mRNA expression was predominantly unchanged. In accordance with mRNA data, mature SP-B was induced significantly by dexamethasone with caffeine (13.8±9.0-fold increase, p = 0.0134). We found a synergistic upregulation of SP-B mRNA expression induced by co-administration of various glucocorticoids and caffeine, achieved by accumulation of intracellular cAMP. This effect was mediated by a caffeine-dependent phosphodiesterase inhibition and by upregulation of both ErbB4 and the GR. These results suggested that caffeine is able to induce the expression of SP-transcription factors and affects the signaling pathways of glucocorticoids, amplifying their effects. Co-administration of caffeine and corticosteroids may therefore be of benefit in surfactant homeostasis.

## Introduction

Pulmonary surfactant is a complex mixture of 90% lipids and 10% proteins lining the inner epithelium of the alveoli, thereby stabilizing the surface of the air-blood barrier and improving gas exchange [1]. The protein fraction of surfactant consists of four different proteins designated as surfactant protein (SP)-A, -B, -C, and -D. The hydrophilic proteins SP-A and SP-D have immunologic properties and are secreted independently from the remaining components of surfactant [2]. After maturation from proproteins, the hydrophobic proteins SP-B and SP-C are stored together with the lipid fraction in the so called lamellar bodies and are then secreted onto the alveolar surface [3]. The major role of these two proteins is to maintain the accurate condition of the lipid surfactant film, although other roles are also discussed [1], [4]. SP-B itself promotes the formation and stability of the surfactant monolayer through its interactions with dipalmitoyl phosphatidylcholine and is mainly synthesized in alveolar type II and Clara cells, the main producers of surfactant [5]. The thyroid transcription factor-1 (TTF-1) has an essential role in the regulation of SP-B transcription [6], while the neuregulin receptor erythroblastic leukemia viral oncogene homolog 4 (ErbB4) has been recently described as a new transcription cofactor for SP-B and, in addition, has a regulatory function which is crucial for fetal lung development [7], [8]. Beside these, various other transcription factors, like jun B proto-oncogene (Jun B), jun proto-oncogene (c-Jun), Sp1 transcription factor (SP1), Sp3 transcription factor (SP3), hepatocyte nuclear factor 3-α (HNF-3α), and cAMP response element binding.

Protein (CREB) have been also implicated in SP-B transcription initiation [6], [9], [10]. The identification of neonates with refractory respiratory failure due to a genetic absence of SP-B highlights the importance of SP-B for surfactant function and the maintenance of lung function [11]. Treatment with surfactant including SP-B has substantially increased survival rates of preterm infants with respiratory distress syndrome (RDS) in the last decades [12], [13].

As methylxanthine, caffeine is used to reduce the frequency of apnea of prematurity [14] without inducing short term side effects [15], thereby facilitates weaning from mechanical ventilation and extubation in very low birth weight infants [16], [17]. Furthermore, methylxanthines can reduce the incidence of bronchopulmonary dysplasia (BPD), most likely by abating the necessity of mechanical ventilation and their anti-inflammatory properties [15], [18]. The variety of actions mediated by methylxanthines are highly dose dependent. At higher plasma concentrations methylxanthines act as unspecific phosphodiesterase (PDE) inhibitors, thereby leading to higher levels of intracellular cyclic adenosine monophosphate (cAMP) [19], [20]. Moreover, their non-specific adenosine receptor antagonism has an impact on neurotransmitter release in the brain, airway smooth muscle constriction in asthmatic patients and many other physiologic mechanisms influenced by the action of adenosine [19], [21]. A recent study also reported a complex relationship between high caffeine levels and pro-inflammatory cytokines [22]. At lower plasma concentrations, a role of methylxanthines in histone acetylation and deacetylation has been hypothesized [19], [23], which could possibly promote SP gene transcription mediated by steroids.

Corticosteroids are given to enhance fetal lung function and maturation [24], thereby affecting the incidence and severity of RDS [25]. Together with caffeine, glucocorticoids are also a therapeutic option for BPD [26]. Glucocorticoids act mainly by binding to the glucocorticoid receptor (GR) and other transcription factors such as NF-κB to activate or inactivate the transcription of various genes [27]. However, potential short- and long term adverse effects of glucocorticoids, especially impairment of growth and neurodevelopment, should be took into account before administration to preterm infants [28].

Taken together, caffeine and glucocorticoids are powerful tools to reduce the incidences of diverse antenatal and postnatal complications in preterm infants such as BPD and RDS [29], [30], and have been extensively used during the last decades. The impact of glucocorticoids on various signaling pathways is well described [31], but consequences of a simultaneous treatment with caffeine are not sufficiently defined yet. Therefore, it is strongly desirable to uncover possible interactions both to prevent harmful side effects or to obtain benefits from these interactions. Since SP-B is the only surfactant-associated protein which is absolutely required for postnatal lung function [5], induction of its transcription and translation is of fundamental importance for the survival of preterm and full-term infants.

The aim of the present study was to investigate the effect of caffeine and different glucocorticoids on the expression of SP-B, since a therapeutic intervention with these two drugs is common during the neonatal period and possible interactions between the different signaling pathways activated are not well described yet.

## Materials and Methods

### Reagents

Caffeine, dexamethasone, betamethasone, prednisone, prednisolone, hydrocortisone, 8-Br-cAMP, rolipram, and RU486 were purchased from Sigma-Aldrich (St. Louis, CA).

### Cells

Airway epithelial cells NCI-H441 (H441) [32], a human lung adenocarcinoma cell line with characteristics of bronchiolar Clara epithelial cells, and A549 cells [33], a human lung carcinoma cell line with characteristics of human alveolar basal epithelial cells, were purchased from ATCC (LGC Standards, Teddington, UK). H441 and A549 cells were cultured in RPMI 1694 and DMEM (Gibco, Life Technologies, Carlsbad, CA), respectively, with additional 5% fetal bovine serum, 100 U/mL penicillin and 100 µg/mL streptomycin (Gibco). Incubation was carried out at 37°C in a humidified atmosphere with 5% CO_2_.

### RNA Extraction and RT-PCR

For RNA extraction, 5×10^5^ H441 or 4×10^5^ A549 cells were seeded on six well plates (Greiner, Frickenhausen, Germany) and grown over night at 37°C. Cells were washed with Dulbecco’s Phosphate Buffered Saline (DPBS) and treated with 1 mL medium containing substances as indicated. After the appropriate time, cells were washed with DPBS and total RNA was isolated using RNeasy Mini Kit (Qiagen, Hilden, Germany) according to the manufacturer’s protocol. Total RNA was eluted in 50 µL Elution Buffer and stored at -80°C until reverse transcription. For RT-PCR, 5 µg of total RNA was reverse transcribed using 1 U M-MuLV Reverse Transcriptase, 100 pmol Oligo(dT)_18_ primer, and 1 mM dNTPs (Fermentas, Thermo Scientific, Waltham, US) in a total volume of 25 µL for 50 min at 42°C. The reaction was terminated by heating at 70°C for 10 min and first strand cDNA was stored at −20°C.

### Quantitative Real Time RT-PCR (qPCR)

For quantitative detection of human SP-B and β-actin mRNA, first strand cDNA was diluted 1 to 10 with deionized H_2_O and 10 µL were analyzed in duplicates of 25 µL reactions using 12.5 µL *Taq*Man® Universal PCR Master Mix (Applied Biosystems, Life Technologies, Carlsbad, CA), 1.5 µL deionized H_2_O, and 1 µL FAM-labeled SP-B (Hs00167036_m1), SP-C (Hs00161628_m1), and β-actin (Hs99999903_m1) probes and primers (Applied Biosystems), respectively. For quantitative detection of human TTF-1, Jun B, c-Jun, Sp1, Sp3, HNF-3α, ErbB4, GR, and GAPDH mRNA, first strand cDNA was diluted as above and 10 µL were analyzed in a 25 µL reaction using 12.5 µL Fast SYBR® Green Master Mix (Applied Biosystems), 0.5 µL deionized H_2_O, and 10 pmol of each forward and reverse primer, respectively. Primers for detection of Jun B, c-Jun, Sp1, Sp3, HNF-3α, ErbB4, GR, and GAPDH mRNA were humJunBfwd 5′-AAAGCCCTGGACGATCTG-3′, humJunBrev 5′-CTGAGGTTGGTGTAAACGG-3′, humc-Junfwd 5′-AACATGCTCAGGGAACAG-3′, humc-Junrev 5′-TCAAGTTCTCAAGTCTGTCTC-3′, humSP1fwd 5′-GCTGTGGGAAAGTGTATGG-3′, humSP1rev 5′-GGCAAATTTCTTCTCACCTG-3′, humSP3fwd 5′-CTACCTTGAATACCAATGACC-3′, humSP3rev 5′-GTACCTCTTCCACCACCT-3′, humHNF-3αfwd 5′-GGAACTGTGAAGATGGAAGGG-3′, humHNF-3αrev 5′-ATGTTGCCGCTCGTAGTC-3′, hErbB4fwd 5′-ATCATCCACACCTAGTCC-3′, hErbB4rev 5′-CATCATTCCCTTAGCTATCTG-3′, hGRfwd 5′-CGTTACCACAACTCACCC-3′, hGRrev 5′-GTGTAAGTTCCTGAAACCTG-3′, hGAPDHfwd 5′-CAAAGTTGTCATGGATGACC-3′, and hGAPDHrev 5′-CCATGGAGAAGGCTGGGG-3′, respectively. Primers for simultaneous detection of both TTF-1 mRNA isoforms have already been described elsewhere [34]. PCRs were performed on an ABI Prism 7500 Sequence Detection System (*Taq*Man®) using a 2-step PCR protocol after initial denaturing of DNA (10 min at 95°C) with 40 cycles of 95°C for 15 s and 60°C for 1 min. In case of measurements including SYBR Green, a melt curve analysis was performed at the end of every run to verify single PCR products. Results of SP-B, ErbB4, GR, and TTF-1 were normalized to β-actin or GAPDH and mean fold changes in mRNA expression were calculated by the ΔΔC_T_ method by Livak and Schmittgen [35].

### Immunoblotting

H441 or A549 cells were rinsed with ice-cold Tris-buffered saline (TBS) and incubated in 100 µL lysis buffer (Cell Lysis Buffer; Cell Signaling Technology, Danvers, MA), 0.1 mM PMSF, Complete Mini Protease Inhibitor Cocktail, and PhosSTOP phosphatase inhibitor cocktail (Roche, Basel, Switzerland) for 10 min on ice. The lysate was cleared by centrifugation at 30,000×g for 10 min, and the supernatant was used for Western immunoblotting analysis. Protein concentrations were determined for each sample using Bradford assay (Bio-Rad, Richmond, CA), and equal amounts of cellular protein were loaded and separated by SDS-PAGE on 10% Bis-Tris gels and electrophoretically transferred to polyvinylidene diﬂuoride membranes (Amersham Pharmacia Biotech, Piscataway, NJ). Membranes were blocked in 5% BSA for 1 h at RT and successively incubated with primary antibodies overnight at 4°C. Western Blots were probed with primary antibodies to SP-B (sc-13978; Santa Cruz Biotechnology Inc., Santa Cruz, CA), TTF-1 (sc-13040; Santa Cruz Biotechnology Inc.), and β-actin (926-42212; LI-COR Inc., Lincoln, NE), followed by staining with corresponding IRDye® secondary antibodies (LI-COR Inc.) for 1 h at RT. Specific protein bands were visualized using an ODYSSEY® Infrared Imaging System (LI-COR Inc.). Accumulated signals were quantifieded using Odyssey Software v2.1 (LI-COR Inc.).

### Immunofluorescence Stainings

1×10^5^ H441 were seeded on 4 well Lab-Tek™ II Chamber Slide™ System (Nunc) and grown over night at 37°C. Cells were washed with DPBS and treated with 1 mL medium containing substances as indicated. After 48 h, cells were fixed and permeabilized with 4% paraformaldehyde and 0.1% TritonX-100 in DPBS for 20 min at room temperature (RT) and 10 min at 4°C, respectively. After blocking with DPBS 5% BSA for 20 min at RT, cells were incubated with 1∶100 dilution of rabbit polyclonal SP-B (Santa Cruz Biotechnology Inc.) antibodies and incubated overnight at 4°C in a humidified chamber. Cells were then washed twice with DPBS, stained with 1∶200 dilution of Alexa Fluor® 488 goat anti-rabbit IgG (Invitrogen, Life Technologies, Carlsbad, CA) for 1 h and 300 nM 4′,6-Diamidin-2-phenylindol (DAPI; Invitrogen) for 4 min at RT, respectively. Slides were washed three times with DPBS and mounted with Fluoromount-G™ (Southern Biotech, Birmingham, AL). Images were captured using a DM IRE 220 microscope (Leica, Solms, Germany).

### Flow Cytometry

For quantification of SP-B protein levels by flow cytometry, H441 cells were seeded and treated as described for RNA extraction, but incubated for 48 h at 37°C. Cells were then detached using 0.05% Trypsin-EDTA (Gibco), washed twice with DPBS, and fixed and permeabilized using Fixation Buffer and Permeabilization Wash Buffer (BioLegend, San Diego, CA), respectively, as specified by the manufacturer. Subsequently, cells were stained as described for immunofluorescence stainings including an additional control using normal rabbit IgG (sc-3888; Santa Cruz Biotechnology Inc.). For staining of GR, a rabbit polyclonal antibody (sc-8992; Santa Cruz Biotechnology Inc.) was used in a 1∶100 dilution (kind gift of Dr. Winfried Neuhaus). Analysis was performed using a FACScan Calibur (Becton Dickinson, Heidelberg, Germany). Data was analyzed using FlowJo software (TreeStar, Ashland, OR).

### Luciferase Assay

5×10^5^ H441 cells were seeded on six well plates (Greiner) and grown over night at 37°C. Cells were then transfected with 3.5 µg of a pGL3-based human SP-B promoter construct pGL3hSP-B (kind gift of Dr. Vijay Boggaram) [36] containing −911/+41 bp of human SP-B 5′ ﬂanking DNA and 5 ng of the *Renilla* luciferase control reporter vector phRL-TK using 6 µg of linear Polyethylenimine MW 25,000 (Polysciences Inc., Warrington, PA) in a total volume of 1 mL of Opti-MEM® (Invitrogen) overnight. Cells were then treated as indicated and after 24 hours luciferase activity was measured by the dual luciferase assay system (Promega Biotech Inc., Madison, WI) according to the manufacturer’s instruction using a Berthold MiniLumat LB 9506 luminometer (Bad Wildbach, Germany). Fireﬂy luciferase activity was normalized by the activity of *Renilla* luciferase under control of the thymidine kinase promoter of phRL-TK. Results are given as relative light units.

### Data Analysis

All results shown are combinations of a minimum of three independent experiments. Results are given as means ± SD. Data were analyzed using students t-Test. A p value ≤0.05 was considered significant. All statistical analyses were performed using Prism® version 5.00 (GraphPad Software, San Diego, CA).

## Results

### Synergistic Effect of Caffeine and Different Glucocorticoids on Expression of Surfactant Protein B mRNA

**Figure 1:**
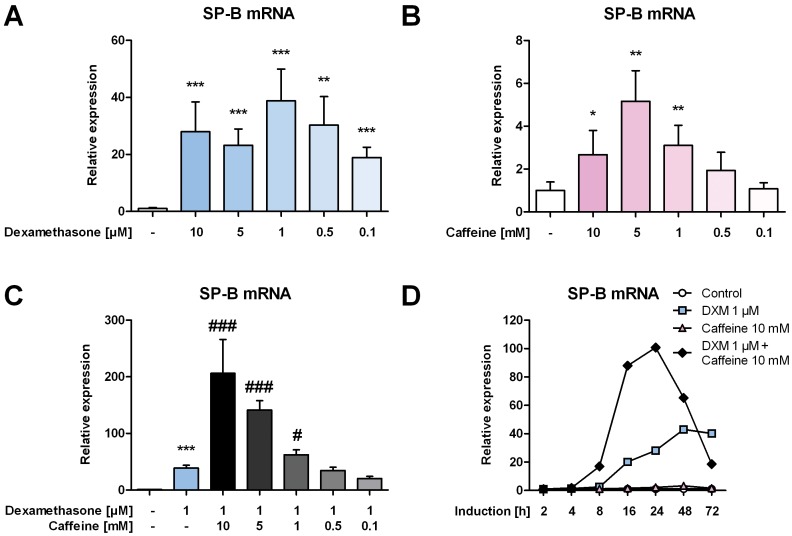
Synergistic effect of caffeine and dexamethasone on expression of SP-B mRNA in H441 cells. H441 cells were treated with different doses of dexamethasone (DXM) (**A**), caffeine (**B**), or combinations (**C**–**D**), and 24 h later(**A**–**C**) or after various timepoints (**D**) qPCR of SP-B mRNA was performed. SP-B mRNA levels were normalized to β-actin, and fold differences compared to untreated cells were calculated. For **A**-**C** means ± SD of at least n = 3 independent experiments are shown. * p<0.05, ** p<0.01 and *** p<0.001 compared to control cells, # p<0.05 and ### p<0.001 compared to cells treated with dexamethasone.

The effect on SP-B mRNA expression of various steroids and caffeine, alone or in combination, was first measured by qPCR. Application of dexamethasone to H441 cells increased SP-B mRNA expression in a dose dependent manner, with highest induction of 38.8±11.1-fold after treatment with 1 µM (Fig. 1A). Increased induction, albeit to a lower extend, was also observed after the administration of 5 mM caffeine to H441 cells in a dose dependent manner leading to a maximal 5.2±1.4-fold increase of SP-B mRNA (Fig. 1B). When dexamethasone and caffeine were given in combination, no additive but rather a synergistic induction of SP-B mRNA expression could be observed. The highest stimulation was achieved using 1 µM dexamethasone together with 10 mM caffeine, leading to 206±59.7-fold significantly higher levels of SP-B mRNA compared to untreated cells and 167±27.4-fold significantly higher levels if compared to cells treated with 1 µM dexamethasone (Fig. 1C). The maximal effect of caffeine and dexamethasone on SP-B mRNA expression was visible after 24 hours (Fig. 1D). In the lung epithelial cell line A549, SP-B mRNA could not or barely be detected. In contrast, SP-B mRNA from A549 cell treated with 1 µM dexamethasone or 10 mM caffeine yielded detectable amounts of SP-B mRNA, although to a much lower level than in comparison to H441 cells (difference >10 C_T_ values). If A549 cells treated with combinations of dexamethasone and caffeine were compared to dexamethasone-treated cells, a synergistic induction of SP-B mRNA of 31.4±11.1-fold could also be observed (Fig. S1).

**Figure 2:**
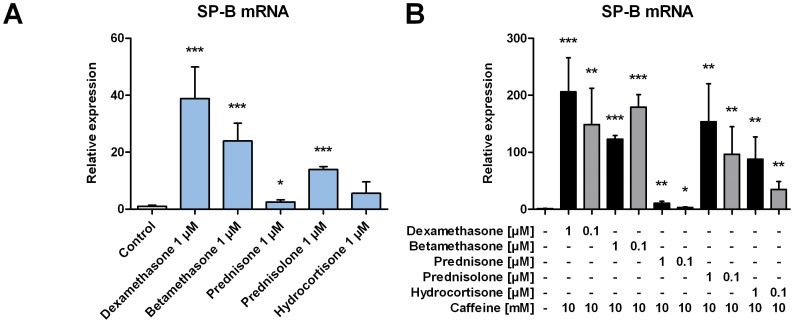
Synergistic effect of caffeine and different glucocorticoids on expression of SP-B mRNA in H441 cells. H441 cells were treated with dexamethasone, betamethasone, prednisone, prednisolone, or hydrocortisone alone (**A**) or in different concentrations in combination with caffeine (**B**). After 24 h qPCR of SP-B mRNA was performed. SP-B mRNA levels were normalized to β-actin, and fold differences compared to untreated cells were calculated. Means ± SD of at least n = 3 independent experiments are shown. * p<0.05, ** p<0.01 and *** p<0.001 compared to control cells.

To test whether the observed effect was restricted to dexamethasone, we also used different steroids. When administered to H441 cells alone, betamethasone determined a 23.9±6.3-fold increase in the expression of SP-B mRNA, followed by a 13.9±1.0-fold increase for prednisolone, 5.6±4.0 for hydrocortisone, and 2.5±0.7 for prednisone (Fig. 2A). When administered in combination with 10 mM caffeine, almost all glucocorticoids induced a synergistic upregulation of SP-B mRNA as for dexamethasone, although to a lower extend. We found a 123±6.4-fold induction of SP-B mRNA for 1 µM betamethasone together with 10 mM caffeine compared to untreated cells, a 154±66.8-fold induction for 1 µM prednisolone, and a 87.9±39.0-fold induction for 1 µM hydrocortisone (Fig. 2B). Prednisone, an inactive form, which is converted to prednisolone by 11β-hydroxysteroid dehydrogenase in the liver [37], failed to mediate the observed synergistic effect (Fig. 2B and Fig. S2).

To identify, whether SP-C is also regulated in a comparable manner by glucocorticoids and caffeine, we investigated SP-C mRNA expression of H441 and A549 cells after induction by qPCR. While changes in SP-C mRNA expression in H441 cells could not be analyzed since a detection was often barely possible for every condition, expression in A549 cells was found to be slightly higher in general, but not regulated (data not shown).

### Enhanced Expression of Surfactant Protein B mRNA After Accumulation of cAMP

**Figure 3:**
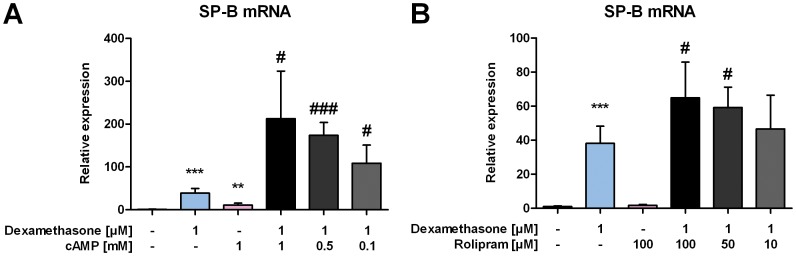
Enhanced expression of SP-B mRNA after accumulation of cAMP. H441 cells were treated as indicated, and 24 h later qPCR of SP-B mRNA was performed. SP-B mRNA levels were normalized to β-actin, and fold differences compared to untreated cells were calculated. (**A**) H441 cells were treated with dexamethasone, cAMP, or different concentrations of cAMP in combination with dexamethasone. (**B**) H441 cells were treated with dexamethasone, the PDE inhibitor rolipram, or different concentrations of rolipram in combination with dexamethasone. Means ± SD of at least n = 3 independent experiments are shown. ** p<0.01 and *** p<0.001 compared to control cells, # p<0.05, ### p<0.001 compared to cells treated with dexamethasone.

At high concentrations, caffeine is known to act as unspecific inhibitor of PDEs. In order to investigate whether this inhibitory function is involved in the observed synergistic upregulation of SP-B mRNA we replaced caffeine with various concentrations of cAMP to mimic caffeine acivity and performed the same experiments as above. After the exposure to 1 mM cAMP and 1 µM dexamethasone, an upregulation of SP-B mRNA comparable to the one achieved with caffeine could be observed in H441 cells (213±111-fold increase). As for caffeine, also this effect was dose dependent (Fig. 3A). Moreover, we also used the PDE4 inhibitor rolipram, which is known to lead to an increased cellular level of cAMP. While the exclusive treatment with rolipram had no significant effect on SP-B mRNA expression compared to untreated cells, we observed a dose-dependent increase of SP-B mRNA expression when rolipram was administered in combination with 1 µM dexamethasone compared to cells exposed to dexamethasone alone (Fig. 3B), which was statistically significant for 100 µM rolipram (p = 0,0251) and 50 µM rolipram (p = 0,0165).

### Influence of Caffeine and Dexamethasone on mRNA Expression of Surfactant Protein B Transcription Factors and SP-B Promoter Activity

**Figure 4:**
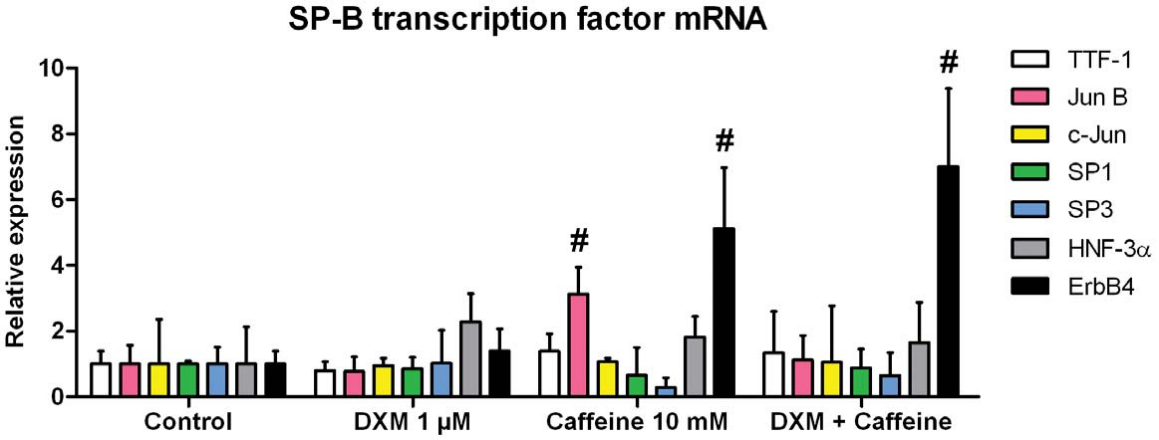
Influence of caffeine and dexamethasone on mRNA expression of SP-B transcription factors. H441 cells were treated with dexamethasone (DXM), caffeine, or combinations and 24 h later qPCR of TTF-1, Jun B, c-Jun, SP1, SP3, HNF-3α, and ErbB4 mRNA was performed. Transcription factor mRNA levels were normalized to GAPDH, and fold differences compared to untreated cells were calculated. Means ± SD of at least n = 3 independent experiments are shown. # p<0.05 compared to cells treated with dexamethasone.

To assess which SP-B transcription factors are involved in the observed upregulatory effect, we measured the mRNA levels of TTF-1, Jun B, c-Jun, SP1, SP3, HNF-3α, and ErbB4 after induction of H441 cells with 1 µM dexamethasone, 10 mM caffeine, or a combination of these drugs. Whereas the transcription of TTF-1, c-Jun, SP1, SP3, and HNF-3α mRNA was not significantly altered by dexamethasone or caffeine alone or in combination, the expression of ErbB4 mRNA was significantly upregulated, being the increase about 5.1±1.9-fold in the presence of 10 mM caffeine (SEM = 1.07, p = 0.0067 compared to untreated cells, and p = 0.0308 compared to dexamethasone-treated cells) (Fig. 4). The addition of 1 µM dexamethasone to cells treated with caffeine further increased ErbB4 mRNA-levels 7.0±2.4-fold above control levels (SEM = 1.36, p = 0.0036 compared to untreated cells, and p = 0.0167 compared to dexamethasone-treated cells), whereas individual treatment with 1 µM dexamethasone had no effect (Fig. 4). A significant upregulation of 3.1±0.8-fold (p = 0.0211) of Jun B mRNA only in case for caffeine-treated cells in comparison to control cells could also be observed. The unaltered expression of TTF-1 after treatment of H441 cells with dexamethasone, caffeine, and combinations was also confirmed at the protein level by Western immunoblotting (Fig. S3). Additionally, we also measured the same SP-B transcription factor mRNAs in A549 cells and could confirm the upregulation of ErbB4 mRNA by caffeine (20.3±16.3-fold increase, p = 0.0295) and also by caffeine in combination with dexamethasone (19.0±12.0-fold increase, p = 0.0102) in comparison to untreated cells (Fig. S4). In contrast to H441 cells, a significant upregulation of TTF-1 mRNA could also be observed by caffeine (3.2±0.8-fold increase, p = 0.0089) and also by caffeine in combination with dexamethasone (13.7±2.5-fold increase, p = 0.0009) in comparison to untreated cells (Fig. S4).

When a promoter construct containing -911/+41 bp of human SP-B 5′ ﬂanking DNA was tested in a Luciferase reporter assay, the synergistic induction observed for simultaneous treatment of H441 cells with dexamethasone and caffeine could not be confirmed for this region of the SP-B promoter. Expression of the Luciferase reporter gene was only modestly, but significantly altered 1.6±0.4-fold (p = 0.0097) after treatment of the cells with 1 µM dexamethasone (Fig. S5).

### Involvement of the Glucocorticoid Receptor in Expression of Surfactant Protein B mRNA

**Figure 5:**
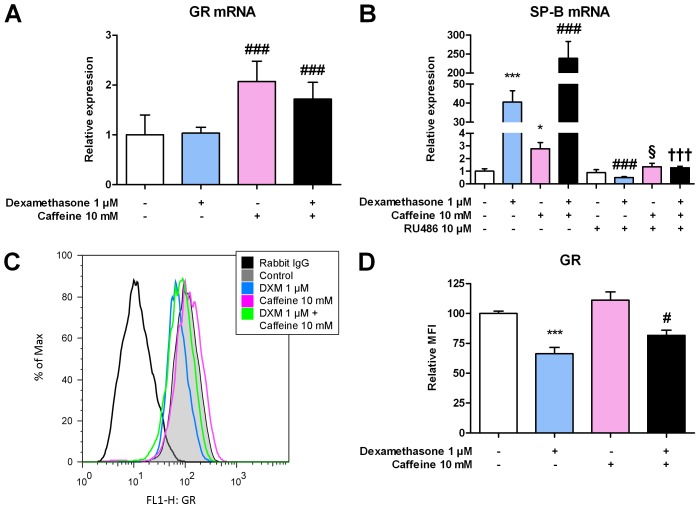
Involvement of the GR in expression of SP-B mRNA. (**A**) H441 cells were treated with dexamethasone, caffeine, or combinations, and 24 h later qPCR of GR mRNA was performed. Measured mRNA levels were normalized to GAPDH, and fold differences compared to untreated cells were calculated. (**B**) H441 cells were treated with dexamethasone, caffeine, the GR antagonist RU486 (mifepristone), or combinations, and 16 h later qPCR of SP-B mRNA was performed. Measured mRNA levels were normalized to β-actin, and fold differences compared to untreated cells were calculated. (**C**, **D**) H441 cells were treated with dexamethasone (DXM), caffeine, or combinations. After 48 h, cells were fixed, staining of the GR was performed, and cells were analyzed by flow cytometry. For **A**, **B** and **D** means ± SD of n = 3 independent experiments are shown. For **C**, a representative image of n = 3 independent experiments is shown. * p<0.05 and *** p<0.001 compared to control cells, ### p<0.001 compared to cells treated with dexamethasone, § p<0.05 compared to cells treated with caffeine, ††† p<0.001 compared to cells treated with dexamethasone and caffeine. MFI, mean fluorescence intensity.

Since the biological effects of glucocorticoids are mediated by their binding to the GR, a ligand-activated transcription factor belonging to the steroid/thyroid hormone receptor superfamily, we investigated whether dexamethasone and caffeine were able to affect GR mRNA expression. We found that the application of 10 mM caffeine to H441 cells increased GR mRNA expression of approximately 2-fold (Fig. 5A) as measured by qPCR. This effect seemed to be unaffected by the addition of 1 µM dexamethasone, since we could not observe any difference in the GR mRNA expression between the untreated or the caffeine-treated cells after its addition. Furthermore, if we added the GR inhibitor RU486 (mifepristone) to dexamethasone and/or caffeine treated cells, the observed increase of SP-B mRNA expression was completely abolished and expression was below or comparable to control levels in all cases (Fig. 5B), indicating an essential role of this receptor in the regulation of SP-B mRNA expression and in the observed synergistic effect of dexamethasone and caffeine.

We also measured GR levels by the flow cytometry. In contrast to mRNA expression, a highly signifcant downregulation of GR expression of about 66% (p<0.001) could be observed after the administration of 1 µM dexamethasone, while no significant modification was detectable after the exposure to 10 mM caffeine (Fig. 5C–D). However, after a combined treatment with dexamethasone and caffeine, the GR expression was partially rescued up to approximately 82% (p = 0.0169) of the GR levels in untreated cells compared to cells treated with dexamethasone (Fig. 5D).

### Influence of Glucocorticoids and Caffeine on Expression of SP-B

In order to investigate if the observed synergistic upregulation of SP-B mRNA by glucocorticoids in combination with caffeine also occurs at the protein level, we performed Western Blot experiments, immunofluorescence stainings, and flow cytometric analyses of dexamethasone, prednisolone and/or caffeine-treated H441 cells. Under normal conditions, H441 cells express only unprocessed or partially processed forms of SP-B (pro-SP-Bs).

**Figure 6:**
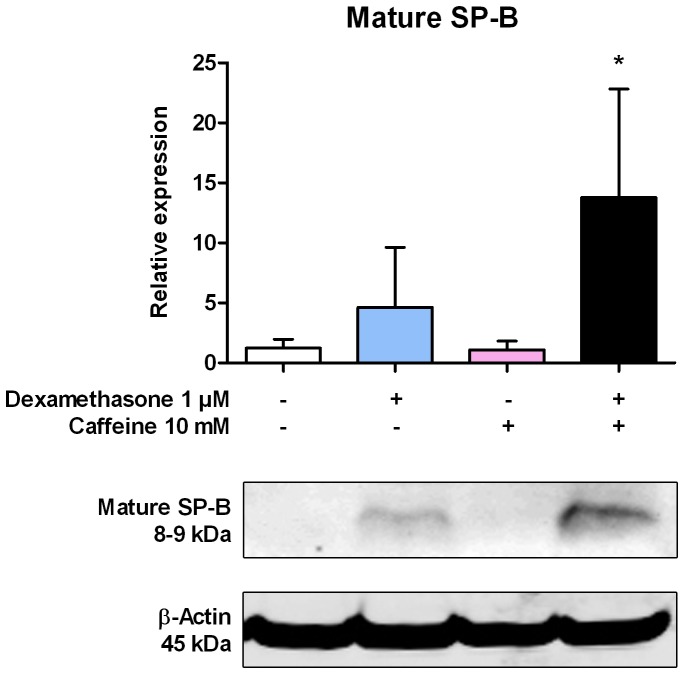
Influence of dexamethasone and caffeine on expression of mature SP-B. H441 cells were treated with 1 µM dexamethasone, 10 mM caffeine, or combinations. After 48 h, cells were lysed and Western immunoblotting analysis against SP-B was performed using a polyclonal antibody. Relative expression levels of SP-B were calculated by normalizing signals to detected β-actin levels. Whereas in untreated cells or cells treated with 10 mM caffeine no mature SP-B could be detected, the expression of mature SP-B in H441 cells was modestly induced by dexamethasone and significantly induced in cells additionally treated with caffeine. Data represent means ± SD of n = 3 independent experiments. * p<0.05 compared to control cells.

To determine, whether dexamethasone, caffeine or the combination of both are influencing the expression of mature SP-B, Western Blot experiments were performed. After 48 h of treatment with dexamethasone, caffeine or combinations, 8–9 kDa mature SP-B could be detected only in cells treated with dexamethasone and cells additionally treated with caffeine, but not in untreated cells or cells exclusively treated with caffeine (Fig. 6). The modest expression of mature SP-B reached 4.6±5.0-fold levels in dexamethasone-treated cells and 13.8±9.0-fold levels (p = 0.0134) in cells treated with dexamethasone in combination with caffeine in comparison to untreated cells (Fig. 6). These results indicate a synergistic effect of steroids and caffeine for mature SP-B also at the protein level.

When immunofluorescence stainings were performed to measure pro- and mature SP-B in total (total SP-B), levels were unaltered 48 hours after the induction with 1 µM dexamethasone (Fig. S6B) or prednisolone (Fig. S6C) in comparison to untreated cells (Fig. S6A). In contrast, after the exposure to 10 mM caffeine, total SP-B levels increased (Fig. S6D). The addition of dexamethasone (Fig. S6E) or prednisolone (Fig. S6F) to caffeine-treated cells did not further increase total SP-B levels. These findings were also confirmed by flow cytometric analyses of total SP-B. Induction of additional total SP-B was only initiated by the presence of caffeine, whereas dexamethasone-treated cells did not differ from untreated cells in total SP-B-content (Fig. S7A–B).

## Discussion

In this study we provide evidence for a synergistic upregulation of SP-B mRNA in type II pneumocytes induced by a combined treatment with glucocorticoids and caffeine. We show that this increase of SP-B mRNA is mediated by an intracellular enrichment of cAMP and is associated with an upregulation of the SP-B transcription cofactor ErbB4 as well as the GR. This synergistic activity mainly affects SP-B mRNA levels and increases mature SP-B, but has, at least in H441 cells, no further impact on the caffeine-mediated increase of total SP-B translation.

An induction of SP-B mRNA by dexamethasone and other glucocorticoids has already been described [38], [39], [40] and could be confirmed in this study. The mechanisms leading to an increase of SP mRNA transcription by glucocorticoids have been already widely discussed and several transcription factors as well as the GR have been implicated in this upregulation [6], [39], [41], [42]. It has also been shown that SP-B mRNA accumulation in H441 cells and explants is a complex interplay between SP-B gene transcription and modification of SP-B mRNA stability and that posttranscriptional factors also influence SP-B expression [39], [43].

Compared to glucocorticoids, we found a slight, dose dependent induction of SP-B mRNA in H441 cells by caffeine alone, which has not been described so far. In combination with dexamethasone and cAMP, isobutylmethylxanthine has been widely used to preserve the phenotypic characteristics of type II epithelial cells in cell culture [44], [45], [46], although an exclusive impact of methylxanthines on the expression of SPs is not well described yet. Considering the ability of caffeine, like many other methylxanthines, to unspecifically inhibit the activity of PDEs, it seems plausible that the observed induction of SP-B mRNA expression by caffeine is mediated by an intracellular enrichment of cAMP, since this effect could be only observed, if high concentrations of caffeine were used. In fact, the observed 10-fold higher level of SP-B mRNA in H441 cells achieved after the exposure to cAMP alone is in consistence with this assumption and with the so far reported role of cAMP as inducer of SP-B mRNA expression [47], [48] and enhancer of GR DNA-binding via protein kinase A [49].

To address, whether the combined treatment of caffeine and glucocorticoids has any further effect on transcription of SP-B mRNA rather than simple additive or reductive effects, we investigated this combined treatment by measuring SP-B mRNA levels. We found, that all metabolic active glucocorticoids induce a strong synergistic upregulation of SP-B mRNA if combined with caffeine. Combinations of dexamethasone, cAMP and isobutylmethylxanthine, a competitive non-selective PDE inhibitor [50], have been described to facilitate differentiation of type II cells from precursor lung epithelial cells and to synergistically induce the transcription of a various set of genes, including SP-A, SP-B and SP-C [43], [46], [51], [52]. By combination of various glucocorticoids with caffeine rather than with cAMP, we could show a comparable synergistic induction of SP-B mRNA as previously observed in cultured human fetal lung epithelial cells using dexamethasone, cAMP and isobutylmethylxanthine [46]. This may indicate a common upregulatory mechanism of caffeine, cAMP and PDE inhibition. By substituting caffeine by cAMP in combination with dexamethasone and without any additional PDE inhibitors we could mimic the previously reported synergistic mRNA induction [46] to the same extend as in combination with caffeine, indicating an essential mediating role of cAMP in this process.

We found that caffeine contributes to the synergism in SP-B mRNA induction only at high, non-physiological doses. Supposing the exclusive role of caffeine as unspecific PDE inhibitor in the context of the above described synergism, a comparable inhibition of PDEs using physiological doses of inhibitors could avoid the usage of high doses of caffeine, thereby eliminating undesirable side effects but maintaining the synergistic mRNA induction, which then could be of benefit in surfactant homeostasis. For this purpose we used the PDE inhibitor rolipram, which is a first generation PDE4 inhibitor characterized by anti-inflammatory and anti-immunomodulatory effects [53]. In fact, a dose dependent, significant increase in SP-B mRNA induction was observed when caffeine was substituted by rolipram. The use of selective PDE inhibitors of the second generation such as the PDE4 inhibitors cilomilast and roflumilast, exhibiting longer half-lives and fewer side effects, may further increase intracellular cAMP levels and thus also potentially therapeutic SP-B levels [54]. The therapeutic potential of PDE4 inhibitors was further demonstrated by their administration to different animal models with lung diseases, in whom they have been shown to improve lung alveolarisation [55] and to attenuate persistent lung injury [56].

To address, which mechanisms are directly responsible for the observed SP-B mRNA upregulation induced by caffeine, we first measured the mRNA levels of TTF-1, the most important transcription factor involved in SP mRNA transcription [57], [58]. However, the reported induction of TTF-1 mRNA expression by dexamethasone and cAMP [46], [52] could be confirmed by qPCR only in A549, but not H441 cells after the treatment with caffeine instead of cAMP. A possible explanation for this disagreement could be the accessibility to different regulatory mechanisms leading to SP gene transcription in fetal or different lung epithelial cell subtypes versus H441 cells, which are derived from adult papillary adenocarcinoma cells, since it is known that other factors play a central role in cell specific activation of SP genes [6]. The hypothesis that glucocorticoids restore TTF-1 and SP-B levels by increasing the TTF-1 autoregulatory mechanism only in hypoplastic fetal lungs supports this possibility [59]. Since it is known that cAMP is able to mediate conformational changes in protein kinase A, which lead to the phosphorylation of TTF-1 [6], [60], an involvement of TTF-1 in the observed SP-B mRNA induction in H441 cells could also be explained by its phosphorylation rather than by its upregulation.

One other recently identified transcriptional cofactor of the SP-B gene is ErbB4 [8]. It has been already associated with the induction of fetal surfactant phospholipid synthesis [61] and seems to have a crucial regulatory role in fetal lung development [62]. We found that caffeine, but not dexamethasone, is able to induce an increase in ErbB4 mRNA in H441 cells and in A549 cells. Therefore, ErbB4 may contribute to the observed caffeine-induced expression of SP-B mRNA and its synergistic induction in combination with dexamethasone. The examination of other SP-B transcription factor mRNAs like Jun B, c-Jun, SP1, SP3, and HNF-3α indicated no involvement in the described synergism.

To directly examine the influence of SP-B transcription factors, we performed SP-B promoter analyses. By this, an approximately 900 bp promoter-region of human SP-B investigated in luciferase assays was not reflecting the same synergistic effects for the reporter gene. These results are indicating a more complex regulation of SP-B mRNA transcription initiation, which may include other factors like cis- or trans-activating elements in combination. Additionally, an increase of SP-B mRNA stability is neither be detectable by the investigation of the SP-B promoter-region. For glucocorticoids this has been previously shown using also H441 cells, where it was postulated that the influence of glucocorticoids on SP-B mRNA expression is more likely due to changes in mRNA stability rather than enhanced transcription [6], [39].

Besides influencing SP-B transcription factors, a modification of the glucocorticoid signal transduction pathway by caffeine could be an explanation for the observed synergistic induction of SP-B mRNA expression by caffeine and glucocorticoids, since it is known that the action of glucocorticoids is mediated by the GR [63]. We found a slight upregulation of GR mRNA after induction with caffeine, which was not modified by the addition of dexamethasone. Some authors already reported an upregulation of both GR mRNA and protein levels by glucocorticoids in various cell lines of lymphoid leukemias [64] and a caffeine-mediated enhancement of GR activity in human osteoblastic cells [65]. Flow cytometric measurement of GR protein levels revealed a downregulation of the GR by dexamethasone, which could be partly restored by the addition of caffeine. This would indicate that caffeine influences the ability of glucocorticoids to increase SP-B mRNA levels by modifying GR expression and therefore possibly reversing the same inhibitory effects of glucocorticoids on SP-B mRNA transcription as shown for SP-A mRNA [66]. Therefore, reversing this inhibitory effect on transcription, the enhanced mRNA stability mediated by glucocorticoids [6] could lead to enormous new amounts of SP-B mRNA, which explains the synergistic induction reported here.

The enhancement of SP-B mRNA by glucocorticoids alone and the synergistic induction by dexamethasone in combination with caffeine was completely abolished by the addition of the GR-inhibitor RU486, which indicates a major involvement of the GR signaling pathway.

When investigating the induction of SP-B by caffeine and glucocorticoids at the protein level, different results were obtained, depending on whether mature or total SP-B (including mature and un- or partially processed forms of SP-B) was investigated. If the mature form of SP-B was analysed by immunoblotting, we found these fully processed form induced by dexamethasone and also significantly to a larger extend by the combination of dexamethasone and caffeine, while no mature SP-B could be detected in untreated cells and cells treated with caffeine alone. This is also in accordance with the observation of previous studies, which claim that SP-B is not fully processed in untreated H441 cells [1]. It also further indicates that parts of the cellular machinery responsible for the final processing of pro-SP-B are inducible by glucocorticoids and, to a higher extent, by their combination with caffeine in H441 cells. When the content of total SP-B was investigated by immunofluorescence and flow cytometry, only a slight but marked increase was obtained by the induction with caffeine. The pro-forms of SP-B are abundantly expressed in H441 cells and may therefore represent static forms which may be affected differently or not affected at all by glucocorticoids in combination with caffeine. Changes of mature SP-B were masked by the detection of the pro-SP-B forms in immunofluorescence stainings. We therefore speculate that caffeine-induced inhibition of PDE and the subsequent increase of cellular cAMP levels are only influencing SP-B mRNA transcription and processing of pro-SP-B forms, but have no impact on caffeine-mediated increase of SP-B translation.

### Conclusion

In conclusion, we described a synergistic upregulation of SP-B mRNA in H441 and A549 cells induced by glucocorticoids in combination with caffeine, which was associated with increased levels of the GR and of the SP-B transcription cofactor ErbB4. Our results indicate that basic SP-B mRNA transcription initiated by glucocorticoids can be potentiated via an increase of cellular cAMP, achieved by a caffeine-mediated inhibition of PDEs. These findings may contribute to our understanding of how the expression of SP genes is regulated. Therefore, the administration of caffeine in combination with glucocorticoids may be of benefit in surfactant homeostasis during the treatment of preterm infants.

## Supporting Information

Figure S1 **Synergistic effect of caffeine and dexamethasone on expression of SP-B mRNA in A549 cells.** A549 cells were treated with 1 µM dexamethasone, 10 mM caffeine, or combinations, and 24 h later qPCR of SP-B mRNA was performed. SP-B mRNA levels were normalized to β-actin, and fold differences compared to cells treated with dexamethasone were calculated. Means ± SD of n = 3 independent experiments are shown. ### p<0.001 compared to cells treated with dexamethasone.
(TIF)

Figure S2 **Chemical structures of different glucocorticoids.** Oxygen is highlighted in red. The 11-keto group of the physiologically inactive prednisone, absent in all other glucocorticoids, is highlighted in green.
(TIF)

Figure S3 **Influence of dexamethasone and caffeine on expression of TTF-1.** H441 cells were treated with different doses of dexamethasone, 10 mM caffeine, or combinations. After 48 h, cells were lysed and Western immunoblotting analysis against TTF-1 was performed using a polyclonal antibody. Relative expression levels of TTF-1 were calculated by normalizing signals to detected β-actin levels.
(TIF)

Figure S4 **Influence of caffeine and dexamethasone on mRNA expression of SP-B transcription factors in A549 cells.** A549 cells were treated with dexamethasone (DXM), caffeine, or combinations and 24 h later qPCR of TTF-1, Jun B, c-Jun, SP1, SP3, HNF-3α, and ErbB4 mRNA was performed. Transcription factor mRNA levels were normalized to GAPDH, and fold differences compared to untreated cells were calculated. Means ± SD of at least n = 3 independent experiments are shown. * p<0.05 and ** p<0.01 compared to control cells, # p<0.05, ## p<0.01 compared to cells treated with dexamethasone.
(TIF)

Figure S5 **Influence of dexamethasone and caffeine on SP-B promoter activity.** H441 cells were transfected with a human SP-B promoter construct containing −911/+44 bp of the human SP-B 5′-ﬂanking DNA linked to a luciferase reporter gene. 16 h later cells were treated with 1 µM dexamethasone, 10 mM caffeine, or combinations, and 24 h later luciferase activity was measured and normalized to the activity of a cotransfected plasmid containing *Renilla* luciferase under control of the thymidine kinase promoter. Only in cells treated with dexamethasone, a significant increase of SP-B promoter activity could be detected. Data represents means ± SD of n = 3 independent experiments measured in duplicates or triplicates. ** p<0.01 compared to untreated cells.
(TIF)

Figure S6 **Influence of glucocorticoids, caffeine and cAMP on expression of total SP-B.** H441 cells were treated with 1 µM dexamethasone, 1 µM prednisolone, 10 mM caffeine, 1 mM cAMP, or combinations. After 48 h, cells were fixed, and immunofluorescence stainings against total SP-B (green) were performed. Nuclei were counterstained with DAPI (blue). Whereas total SP-B expression in untreated cells was low (**A**), the treatment with caffeine induced an increased expression (**D**). Simultaneous treatment with either dexamethasone (**E**) or prednisolone (**F**) had no additional effect. Individual treatment with either dexamethasone (**B**) or prednisolone (**C**) did not increase total SP-B levels in comparison to untreated cells. Representative images of n = 3 independent experiments are shown.
(TIF)

Figure S7 **Flow cytometric analysis of total SP-B in H441 cells treated with dexamethasone and/or caffeine.** H441 cells were treated with 1 µM dexamethasone (DXM), 10 mM caffeine, or combinations and 48 h later cells were fixed, staining of total SP-B was performed, and total SP-B levels were measured by flow cytometry. (**A**) Representative histogram of n = 3 independent experiments. (**B**) Mean fluorescence intensity (MFI) values for total SP-B are shown as means ± SD of n = 3 independent experiments. * p<0.05 compared to control cells.
(TIF)
