# Supplementary figures and images for: Synergistic Effect of Caffeine and Glucocorticoids on Expression of Surfactant Protein B (SP-B) mRNA
Source: PLoS One. 2012 Dec 14;7(12):e51575. doi: 10.1371/journal.pone.0051575 (PMC3522739; doi:10.1371/journal.pone.0051575)

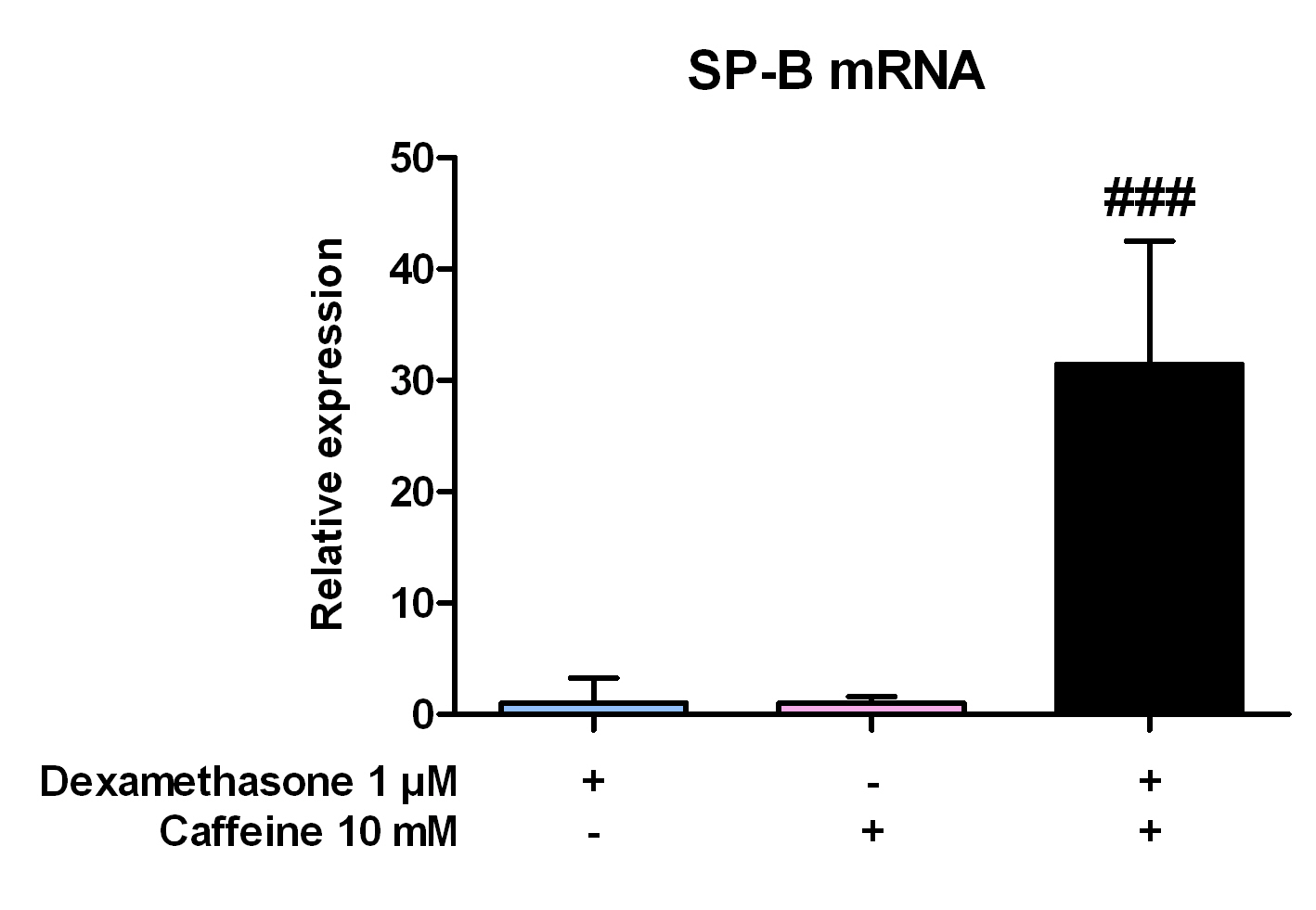

Supplement: Figure S1 — Synergistic effect of caffeine and dexamethasone on expression of SP-B mRNA in A549 cells. A549 cells were treated with 1 µM dexamethasone, 10 mM caffeine, or combinations, and 24 h later qPCR of SP-B mRNA was performed. SP-B mRNA levels were normalized to β-actin, and fold differences compared to cells treated with dexamethasone were calculated. Means ± SD of n = 3 independent experiments are shown. ### p<0.001 compared to cells treated with dexamethasone. (TIF) [file pone.0051575.s001.tif]

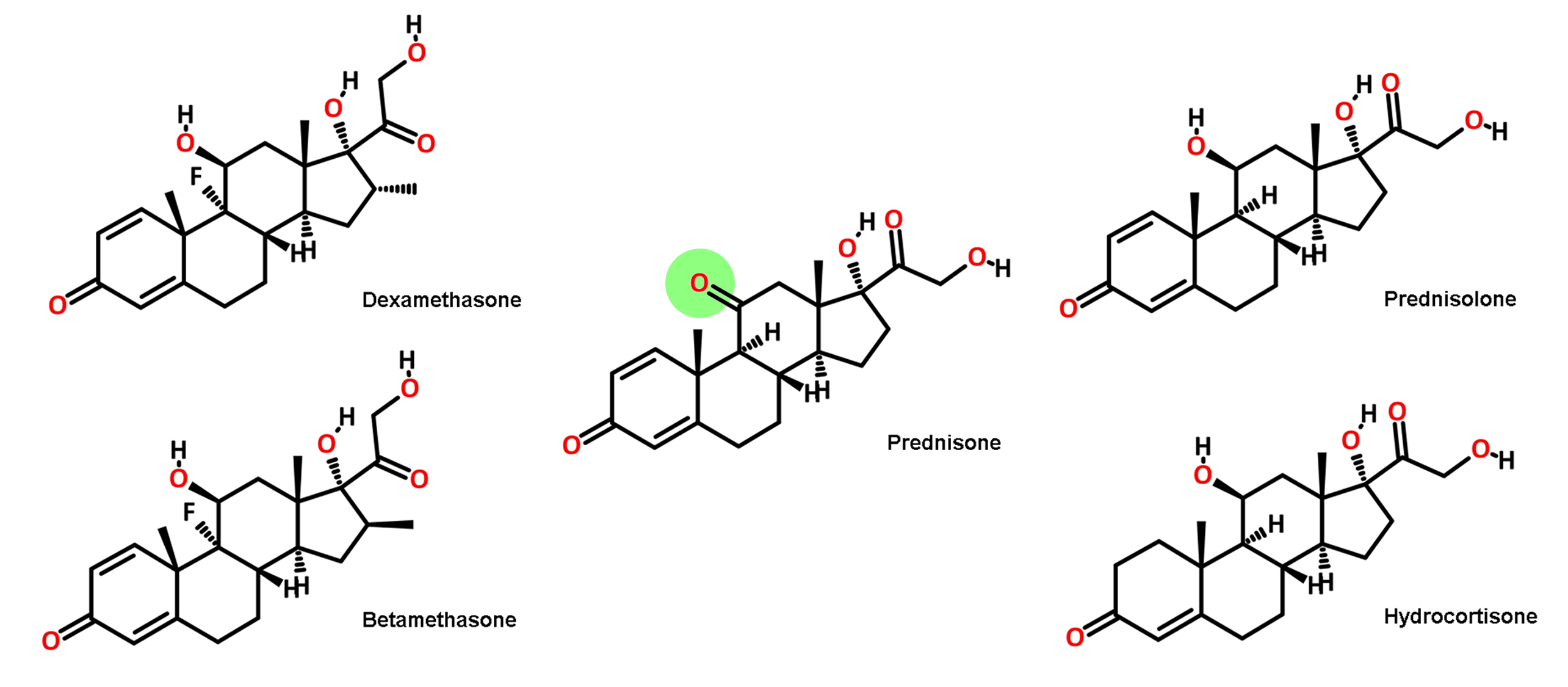

Supplement: Figure S2 — Chemical structures of different glucocorticoids. Oxygen is highlighted in red. The 11-keto group of the physiologically inactive prednisone, absent in all other glucocorticoids, is highlighted in green. (TIF) [file pone.0051575.s002.tif]

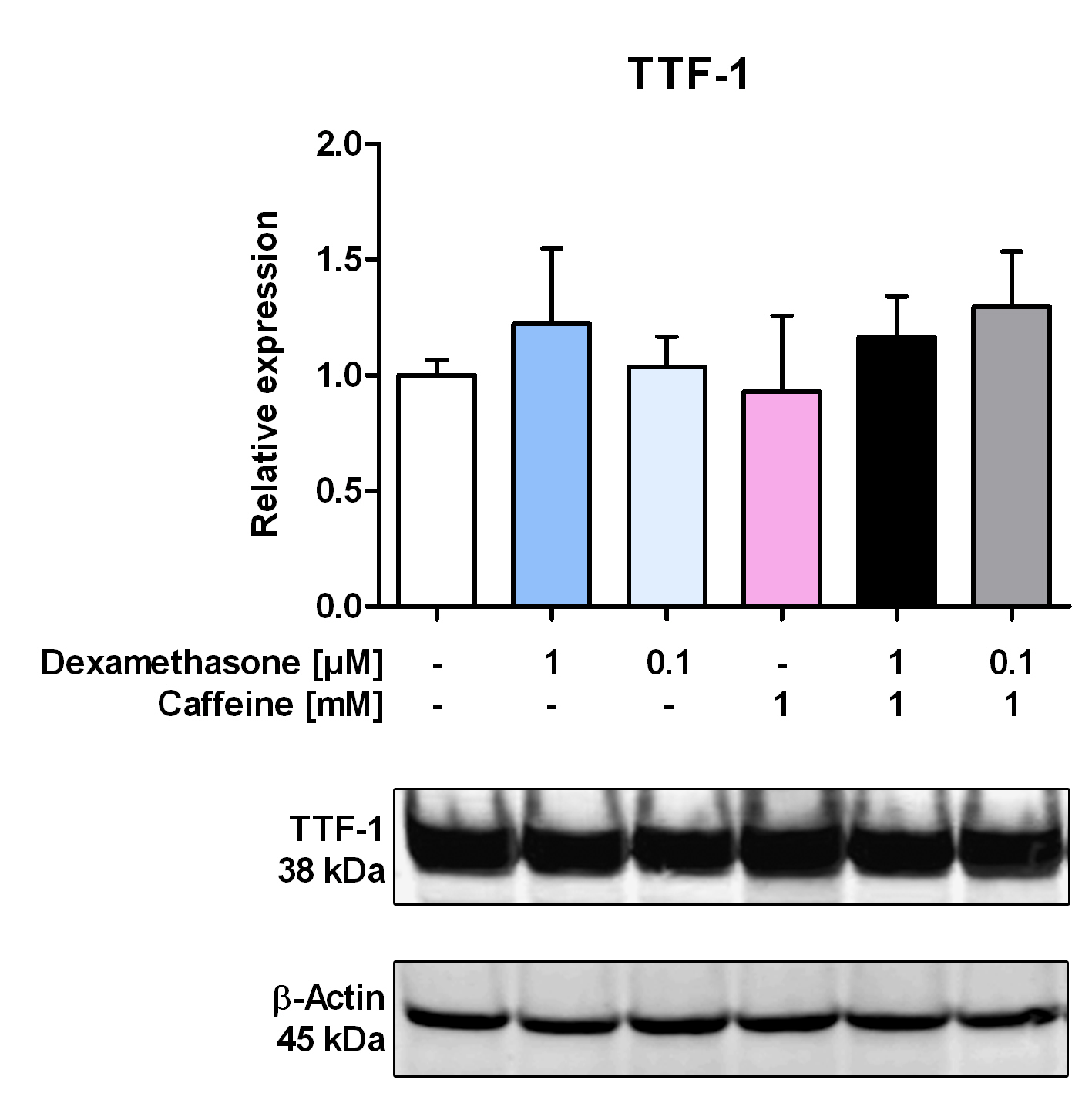

Supplement: Figure S3 — Influence of dexamethasone and caffeine on expression of TTF-1. H441 cells were treated with different doses of dexamethasone, 10 mM caffeine, or combinations. After 48 h, cells were lysed and Western immunoblotting analysis against TTF-1 was performed using a polyclonal antibody. Relative expression levels of TTF-1 were calculated by normalizing signals to detected β-actin levels. (TIF) [file pone.0051575.s003.tif]

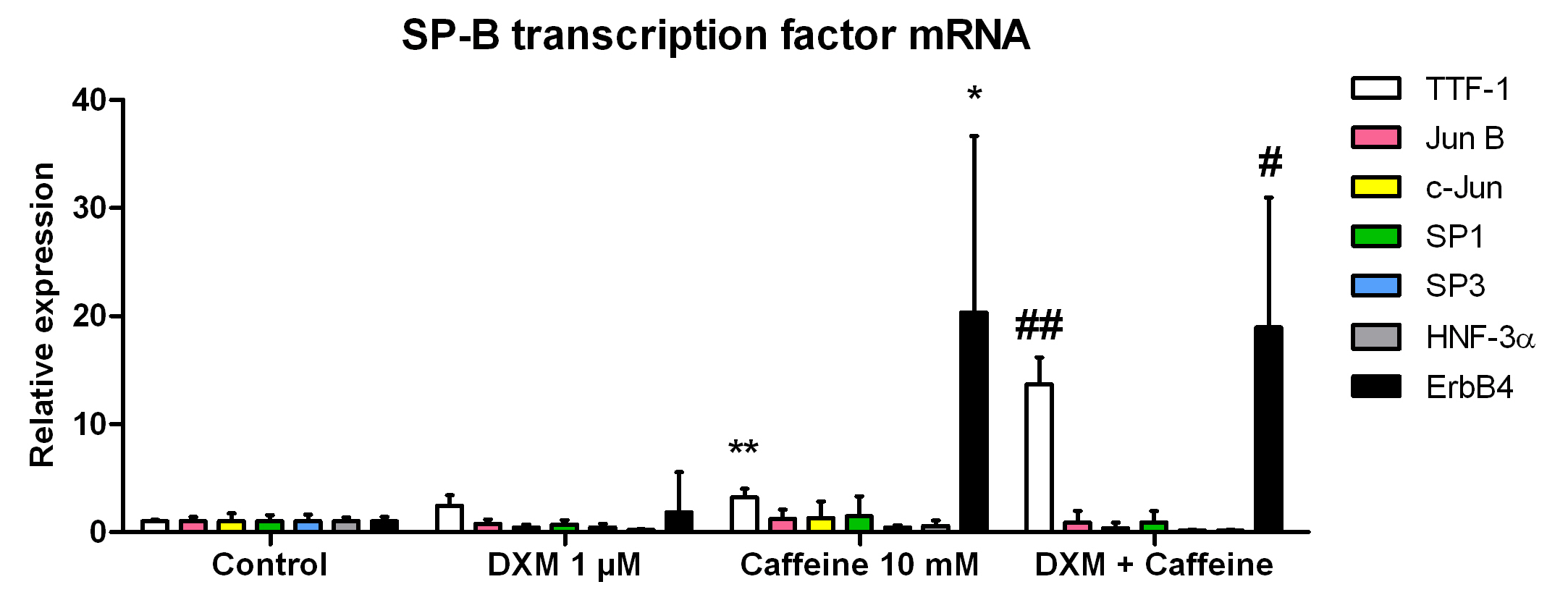

Supplement: Figure S4 — Influence of caffeine and dexamethasone on mRNA expression of SP-B transcription factors in A549 cells. A549 cells were treated with dexamethasone (DXM), caffeine, or combinations and 24 h later qPCR of TTF-1, Jun B, c-Jun, SP1, SP3, HNF-3α, and ErbB4 mRNA was performed. Transcription factor mRNA levels were normalized to GAPDH, and fold differences compared to untreated cells were calculated. Means ± SD of at least n = 3 independent experiments are shown. * p<0.05 and ** p<0.01 compared to control cells, # p<0.05, ## p<0.01 compared to cells treated with dexamethasone. (TIF) [file pone.0051575.s004.tif]

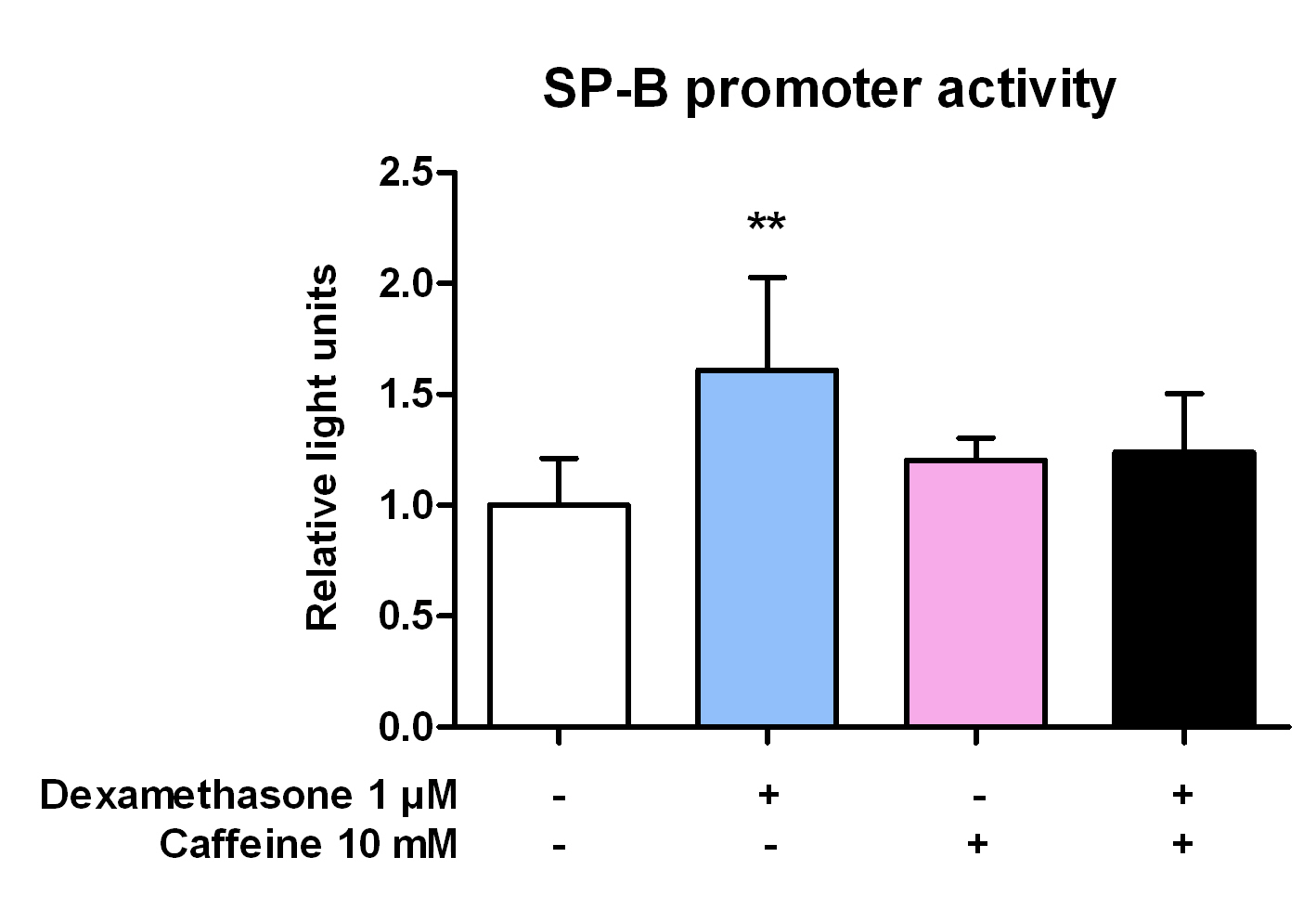

Supplement: Figure S5 — Influence of dexamethasone and caffeine on SP-B promoter activity. H441 cells were transfected with a human SP-B promoter construct containing −911/+44 bp of the human SP-B 5′-ﬂanking DNA linked to a luciferase reporter gene. 16 h later cells were treated with 1 µM dexamethasone, 10 mM caffeine, or combinations, and 24 h later luciferase activity was measured and normalized to the activity of a cotransfected plasmid containing Renilla luciferase under control of the thymidine kinase promoter. Only in cells treated with dexamethasone, a significant increase of SP-B promoter activity could be detected. Data represents means ± SD of n = 3 independent experiments measured in duplicates or triplicates. ** p<0.01 compared to untreated cells. (TIF) [file pone.0051575.s005.tif]

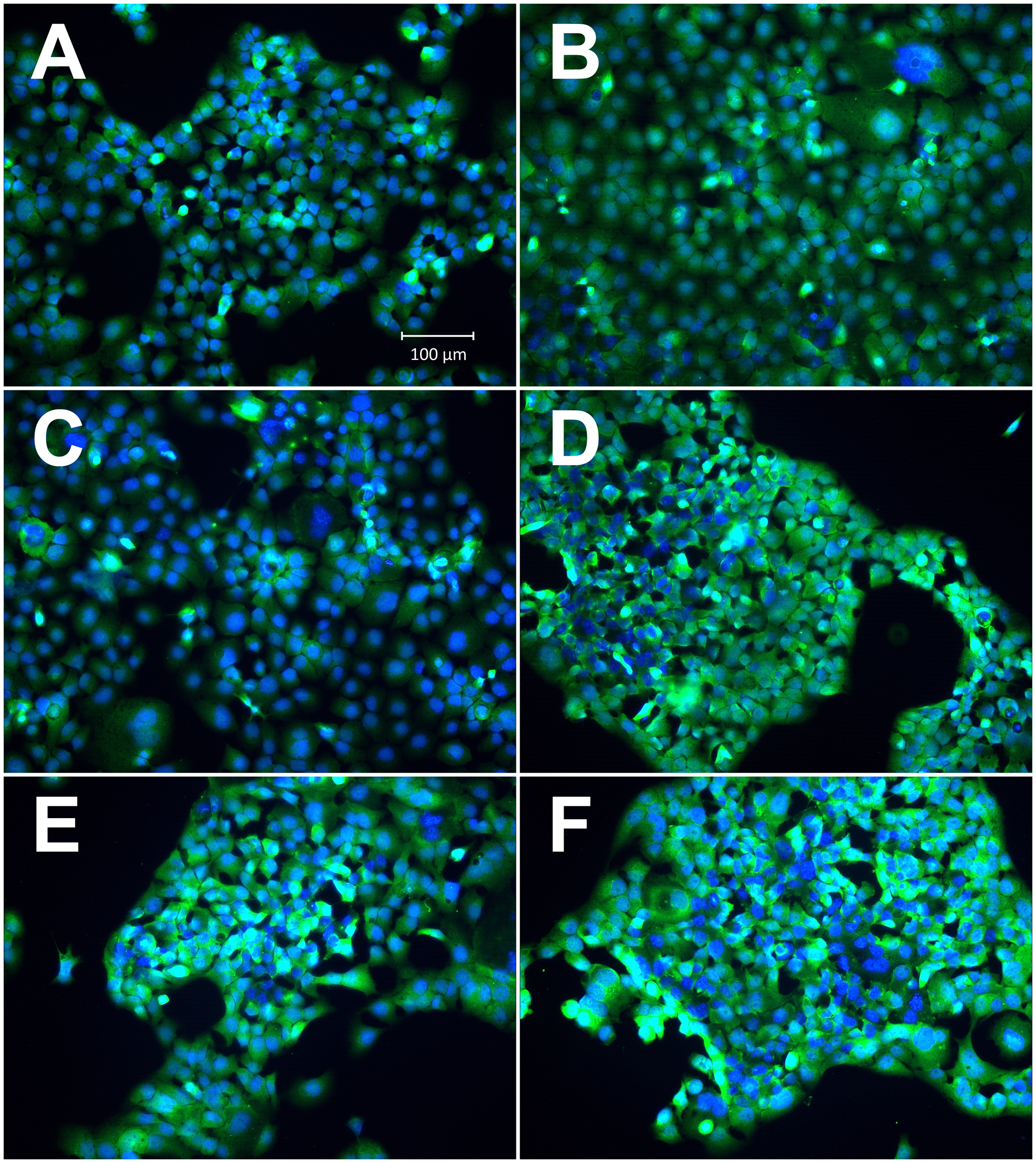

Supplement: Figure S6 — Influence of glucocorticoids, caffeine and cAMP on expression of total SP-B. H441 cells were treated with 1 µM dexamethasone, 1 µM prednisolone, 10 mM caffeine, 1 mM cAMP, or combinations. After 48 h, cells were fixed, and immunofluorescence stainings against total SP-B (green) were performed. Nuclei were counterstained with DAPI (blue). Whereas total SP-B expression in untreated cells was low (A), the treatment with caffeine induced an increased expression (D). Simultaneous treatment with either dexamethasone (E) or prednisolone (F) had no additional effect. Individual treatment with either dexamethasone (B) or prednisolone (C) did not increase total SP-B levels in comparison to untreated cells. Representative images of n = 3 independent experiments are shown. (TIF) [file pone.0051575.s006.tif]

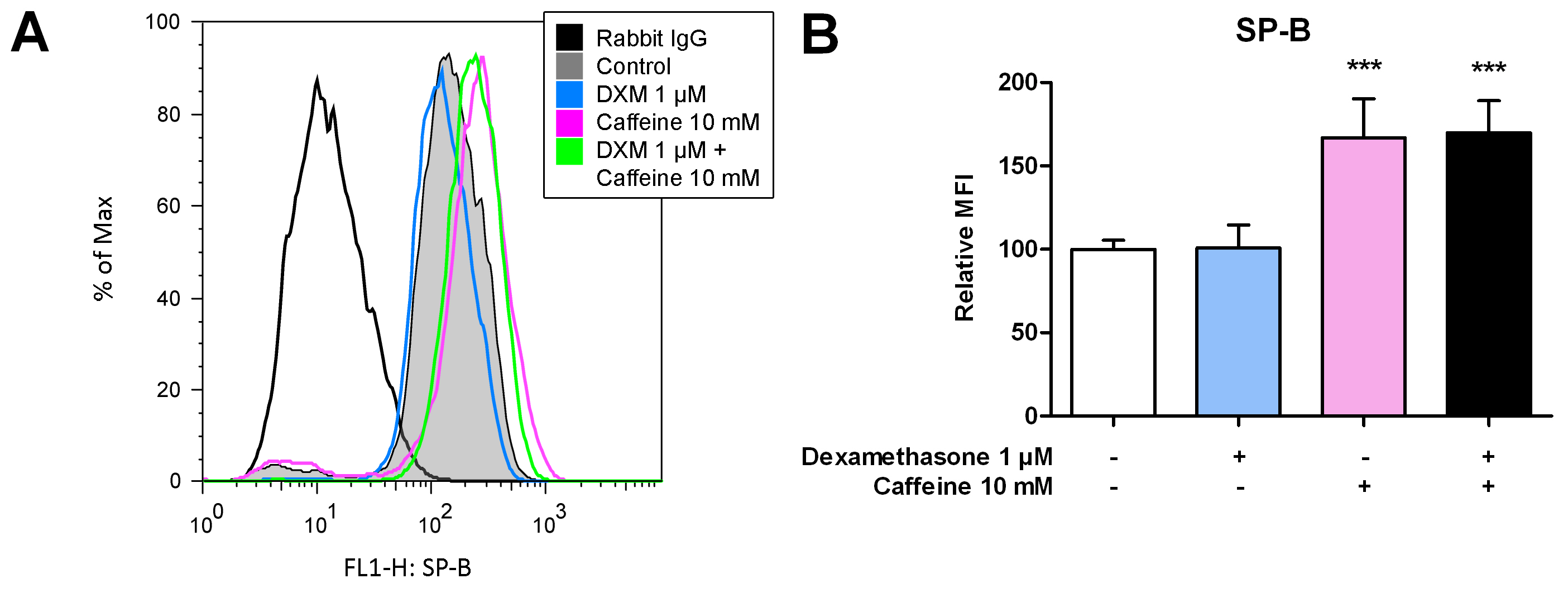

Supplement: Figure S7 — Flow cytometric analysis of total SP-B in H441 cells treated with dexamethasone and/or caffeine. H441 cells were treated with 1 µM dexamethasone (DXM), 10 mM caffeine, or combinations and 48 h later cells were fixed, staining of total SP-B was performed, and total SP-B levels were measured by flow cytometry. (A) Representative histogram of n = 3 independent experiments. (B) Mean fluorescence intensity (MFI) values for total SP-B are shown as means ± SD of n = 3 independent experiments. * p<0.05 compared to control cells. (TIF) [file pone.0051575.s007.tif]
